# The 2021 World Health Organization Central Nervous System Tumor Classification: The Spectrum of Diffuse Gliomas

**DOI:** 10.3390/biomedicines12061349

**Published:** 2024-06-18

**Authors:** Racine Gue, Dhairya A. Lakhani

**Affiliations:** 1 Department of Neuroradiology, West Virginia University, Morgantown, WV 26506, USA; 2 Department of Radiology and Radiological Sciences, Johns Hopkins University, Baltimore, MD 21218, USA

**Keywords:** WHO classification, brain tumor, glioma

## Abstract

The 2021 edition of the World Health Organization (WHO) classification of central nervous system tumors introduces significant revisions across various tumor types. These updates, encompassing changes in diagnostic techniques, genomic integration, terminology, and grading, are crucial for radiologists, who play a critical role in interpreting brain tumor imaging. Such changes impact the diagnosis and management of nearly all central nervous system tumor categories, including the reclassification, addition, and removal of specific tumor entities. Given their pivotal role in patient care, radiologists must remain conversant with these revisions to effectively contribute to multidisciplinary tumor boards and collaborate with peers in neuro-oncology, neurosurgery, radiation oncology, and neuropathology. This knowledge is essential not only for accurate diagnosis and staging, but also for understanding the molecular and genetic underpinnings of tumors, which can influence treatment decisions and prognostication. This review, therefore, focuses on the most pertinent updates concerning the classification of adult diffuse gliomas, highlighting the aspects most relevant to radiological practice. Emphasis is placed on the implications of new genetic information on tumor behavior and imaging findings, providing necessary tools to stay abreast of advancements in the field. This comprehensive overview aims to enhance the radiologist’s ability to integrate new WHO classification criteria into everyday practice, ultimately improving patient outcomes through informed and precise imaging assessments.

## 1. Introduction

Since its inception in 1956, the WHO Classification of Tumors, commonly referred to as the WHO Blue Books, has evolved from a simple list of terms to a comprehensive resource that integrates diverse information including histological, immunohistochemical, genetic, epidemiological, and clinical data [1,2]. This series has become the global standard, essential for establishing uniform nomenclature for human cancers. Each edition has expanded to reflect the growing understanding of cancer biology, employing a systematic approach to the multifaceted nature of tumor classification [3]. Tumor types are meticulously detailed based on their localization, clinical features, epidemiology, etiology, pathogenesis, histopathology, diagnostic molecular pathology, staging, prognosis, and prediction [4]. This detailed and systematic approach is crucial for radiologists to accurately help diagnose, stage, and predict tumor behavior, ensuring consistent and informed patient care.

In this review, we aim to present the evolution of the latest updates on adult diffuse gliomas, highlighting the most relevant aspects for radiologists, along with genetic information that aids in understanding their classification, behavior, and imaging findings. Additionally, we provide pertinent information on some newly classified entities and pediatric-type diffuse high-grade gliomas, recognizing the overlap between these categories.

## 2. Significance and Context

The standardized terminology adopted by the WHO Blue Books has profound implications across various medical disciplines, in the case of CNS Tumors, enhancing communication among multidisciplinary teams that include neurosurgeons, neurologists, neuro-oncologists, neuropathologists, and neuroradiologists [5]. For neuroradiologists, this consistent language is critical, as it ensures that imaging findings are interpreted in alignment with global standards, enabling accurate correlations with clinical and pathological data [5]. This facilitates seamless collaboration in treatment planning and decision-making, allowing for clear discussions about tumor types, subtypes, and characteristics [6,7].

Furthermore, the use of standardized terminology in the classification supports accurate data collection for population-based cancer registries [5]. This meticulous approach empowers researchers to conduct comprehensive analyses of histological and genetic variations, thus enriching our understanding of cancer epidemiology and informing the development of targeted public health interventions [5,8].

Additionally, the advocacy for standardized terminology by the WHO Blue Books promotes consistency in documentation practices. This ensures precise recording and reporting of CNS tumor cases across healthcare systems and registries, enhancing data integrity and facilitating comparative analyses [5]. Such detailed documentation not only supports the accurate representation of clinical cases, but also underpins research efforts aimed at unraveling tumor biology, identifying prognostic factors, and advancing innovative therapeutic strategies [3,5,6,7].

This comprehensive approach helps maintain the highest standards of clinical practice and research.

### 2.1. Innovation in Molecular Diagnostics Prompts Necessary Revisions

The landscape of CNS tumor classification has dramatically transformed with the integration of molecular medicine techniques, marking a significant departure from the traditional reliance solely on histological findings. Historically, the foundation of tumor classification has been built on histological examination, utilizing tools such as light microscopy, immunohistochemistry, and various tissue staining methods. These techniques have provided essential insights into the cellular characteristics and structures of tumors, facilitating initial diagnostic assessments [1].

However, the last century has witnessed the introduction of groundbreaking technologies, notably DNA and RNA sequencing, which have revolutionized the approach to tumor diagnosis and classification. These molecular methods allow for a more detailed analysis of the genetic and molecular blueprints of tumors, offering a deeper understanding of their origins and behaviors [3,9].

Among the most significant recent advances is methylome profiling, a technique that analyzes DNA methylation patterns across the entire genome [10]. Over the past decade, methylome profiling has emerged as a critical tool for CNS tumor classification [11]. It has proven especially adept at identifying a wide range of tumor types and subtypes, providing a level of precision that surpasses traditional methods [10,12]. This technology not only enhances the accuracy of tumor classification, but also proves invaluable in diagnosing complex, challenging neoplasms and rare tumor forms that might elude conventional diagnostic approaches [11,13].

While the availability of methylome profiling is not yet universal due to its associated technological and cost considerations, the advantages it brings are undeniable [4,12]. This technique enhances diagnostic precision and specificity significantly, setting the stage for more tailored therapeutic approaches and more accurate prognostic assessments [13]. As the accessibility of this technology improves, it promises to refine the diagnostic landscape of CNS tumors further [13]. With more detailed molecular profiles at their disposal, clinicians can offer more personalized and effective care to patients, ensuring that treatments are well-aligned with the unique genetic makeup of each tumor [9].

### 2.2. Overview of Updates

#### 2.2.1. Rising Importance of Molecular Diagnostics

This evolution in diagnostic capabilities was utilized in the 2016 WHO classification of CNS tumors, which marked a significant shift by incorporating molecular markers into tumor classification [14]. This integration allowed for a more precise identification of tumor subtypes, leveraging cutting-edge molecular diagnostics to enhance classification accuracy [4]. The 2021 update to the WHO classification built on this foundation, capitalizing on the broader availability of advanced diagnostics to expand their use further [12]. This ongoing development in CNS tumor standards continues to improve the alignment between clinical practice and the latest scientific insights, ensuring that patient care keeps pace with technological advancements in the field [7,9,15].

Furthermore, molecular markers now play a pivotal role in both the grading and prognosis estimation of various tumors [16]. For example, cyclin-dependent kinase inhibitor A/B (CDKN2A/B) homozygous deletion in isocitrate dehydrogenase (IDH)-mutant astrocytomas and the presence of telomerase reverse transcriptase (TERT) promoter mutations, epidermal growth factor receptor (EGFR) amplification, and +7/−10 chromosomal copy-number changes in the IDH-wildtype glioblastoma are critical for accurate classification and prognosis [16,17]. These molecular parameters take precedence over histological findings when assigning a tumor grade [9,14].

As our understanding of the molecular underpinnings of CNS tumors deepens, the task of classifying these tumors becomes increasingly intricate. While some tumors fit neatly into newly established molecular classifications, others align inconsistently or not at all, prompting an evolving hybrid approach to taxonomy [3]. This transition reflects the dynamic nature of the current landscape and reinforces a shift towards a future classification system that may rely more heavily on molecular diagnostics than traditional methods [3,4].

#### 2.2.2. Update: Not Otherwise Specified and Not Elsewhere Classified

The 2021 WHO classification acknowledges these complexities by introducing categories like “Not Otherwise Specified” (NOS) and “Not Elsewhere Classified” (NEC) [18,19]. These categories serve to differentiate between well-characterized WHO diagnoses and those cases where diagnostic or molecular information is lacking or inconclusive. The designation of “NOS” is used when specific criteria for a definitive WHO diagnosis are not met due to the absence or incomplete nature of molecular analysis. This indicates that while some diagnostic efforts have been made, they do not provide enough information to precisely categorize the tumor under existing WHO classifications [18,19].

Conversely, the “NEC” category is applied when, despite comprehensive diagnostic efforts, including successful pathological and molecular testing, the tumor does not fit any established WHO diagnostic criteria. This suggests that the tumor presents unique or atypical features that do not conform to the current understanding [18,19].

#### 2.2.3. Update: Types and Subtypes

Continuing its historical commitment to systematic organization, the 2021 WHO classification updates strive to standardize tumor diagnosis across various organ systems, enhancing consistency, and clarity [1,3]. This iteration incorporates advanced molecular insights gained from recent technological progress, structuring the classification by starting with the tumor’s site of origin [3]. From there, tumors are grouped based on shared characteristics, and are further categorized at the family or class level. Subsequent divisions into “types” and “subtypes” provide more detail, reflecting distinctions based on molecular and histopathological features [12]. Notably, the terminology has evolved, transitioning from “entity” to “type” and from “variant” to “subtype”, a change that better captures the heterogeneity of tumors and emphasizes the increasing role of molecular details in their classification [3,10].

#### 2.2.4. Update: Tumor Grading

With these updates, the grading system for CNS tumors has also undergone significant changes, including the adoption of Arabic numerals instead of Roman numerals [10]. This adjustment aims to minimize typographical errors and ensure consistency across different organ systems [10]. Furthermore, while previous WHO classifications applied a uniform grading system across various CNS tumor types, the 2021 WHO classification of CNS tumors introduces a within-type grading approach [10]. This method offers greater flexibility and emphasizes the biological similarities within specific tumor types. Such a targeted approach aligns CNS tumor grading more closely with the grading systems used for neoplasms in other organ systems [2,10]. Despite these extensive revisions, the term “CNS WHO Grade” remains unchanged, serving as a consistent marker to denote the assigned grade within the updated classification system [3].

#### 2.2.5. Update: Continued Integration of Histological and Molecular Markers

The 2021 WHO classification of CNS tumors introduces new types and updates the nomenclature to clarify molecular features, aligning it with the cIMPACT guidelines [20]. The updates in these areas are outlined below, reflecting the latest scientific understanding and aiming to enhance diagnostic precision and treatment approaches [4,12].

### 2.3. Broad Categorization Overview

The 2021 WHO classification of CNS tumors organizes tumors into several broad categories, including gliomas, glioneuronal, and neuronal tumors; choroid plexus tumors; embryonal tumors; pineal tumors; cranial and paraspinal nerve tumors; meningiomas; mesenchymal, non-meningothelial tumors involving the CNS; melanocytic tumors; hematolymphoid tumors involving the CNS; germ cell tumors; tumors of the sellar region; metastases to the CNS; and genetic tumor syndromes involving the CNS [3,4].

#### 2.3.1. Gliomas, Glioneuronal Tumors and Neuronal Tumors

The chapter dedicated to gliomas, glioneuronal, and neuronal tumors covers a substantial portion of primary intra-axial CNS tumors [3,12]. Within this chapter, these tumors are further classified into six distinct families, a division that reflects their significant differences in underlying biology, growth patterns, and typical patient demographics [12,14].

#### 2.3.2. Update: Gliomas, Glioneuronal Tumors and Neuronal Tumors

A notable refinement in the latest edition involves distinguishing gliomas primarily occurring in adults from those typically found in children [9,14]. This is further propelled by heightened molecular insights [14]. For instance, within pediatric-type diffuse gliomas, the classification now encompasses specific molecular subtypes such as Diffuse pediatric-type high-grade glioma, categorized as either H3-wildtype or IDH-wildtype, and Diffuse hemispheric glioma, characterized by H3 G34-mutation. Furthermore, the transition from “H3 K27-mutant” to “H3 K27-altered” within pediatric gliomas marks a significant development, indicative of deeper understandings into the genetic mechanisms underpinning these tumors [3,20,21].

While this review primarily delves into adult-type diffuse gliomas, it is essential to briefly mention Diffuse Midline Glioma, H3 K27-altered and newly recognized tumors due to overlapping imaging features and affected populations [22]. These include Diffuse Hemispheric Glioma, H3 G34-mutation, Diffuse Pediatric-Type High-Grade Glioma, H3-wildtype or IDH-wildtype, and High-Grade Astrocytoma with Piloid Features [14]. These examples highlight the expanding spectrum of gliomas and underscore the importance of comprehensive diagnostic approaches across age groups.

#### 2.3.3. Adult-Type Diffuse Gliomas

Adult-type diffuse gliomas have been significantly simplified in the 2021 WHO classification, going from fifteen entities to just three: Astrocytoma, IDH-mutant; Oligodendroglioma, IDH-mutant and 1p/19q codeleted; and Glioblastoma, IDH-wildtype. Grading within these types now considers molecular markers alongside histology [3].

## 3. Astrocytoma, IDH-Mutant

### 3.1. Epidemiology and Localization

IDH-mutant astrocytoma, often arising in adults aged thirty to forty, predominantly develops in the frontal lobes, but can occur throughout the central nervous system, including the cerebral hemispheres, brainstem, and spinal cord [23].

### 3.2. Brief Genetic Overview

The most defining feature of IDH-mutant astrocytoma is the mutation in either the IDH1 or IDH2 gene [23]. IDH1 mutations are more common, especially the R132H mutation [24,25]. These mutations are critical because they result in the production of an abnormal enzyme that converts a normal cellular metabolite, alpha-ketoglutarate, into an abnormal product, 2-hydroxyglutarate (2-HG) [26]. This byproduct is not typically found in high concentrations in normal cells and has significant implications for tumor biology [27,28]. The accumulation of 2-HG in tumor cells is particularly harmful because it mimics the shape and function of alpha-ketoglutarate but disrupts normal processes [29]. 2-HG is a competitive inhibitor of several alpha-ketoglutarate-dependent dioxygenases, which are crucial for proper cell function [30]. These enzymes include histone demethylases and the Ten-Eleven Translocation (TET) enzymes involved in DNA demethylation [29,30]. By inhibiting these enzymes, 2-HG causes widespread changes in the methylation landscape of both histones (proteins around which DNA is wrapped) and DNA itself [30]. This interference results in the abnormal methylation or “silencing” of DNA in certain regions [28,31]. In turn, this abnormal methylation changes the activity of genes in ways that can promote tumor growth [32,33,34,35].

In addition to IDH mutations, astrocytomas often exhibit other genetic alterations, including tumor protein p53 (TP53) and alpha-thalassemia/mental retardation, and X-linked (ATRX) mutations [35,36]. TP53 mutations compromise the protein’s ability to regulate cell growth and initiate apoptosis, thereby allowing cells with damaged DNA to continue dividing [37]. ATRX mutations disrupt chromatin remodeling, which is crucial for DNA replication and repair, and are associated with a phenotype that enables cancer cells to maintain telomere length and avoid senescence, contributing to their classification as CNS WHO grades 2–4 [7,37,38,39].

#### Relevance

The IDH mutation serves as a critical diagnostic and prognostic marker [33]. Testing for IDH status is now a standard part of the pathological assessment of gliomas [24]. Specific inhibitors targeting the mutant IDH enzymes are in development and clinical trials, offering a new avenue for targeted therapy in managing these tumors [31].

### 3.3. Update

A notable change in the 2021 WHO CNS classification involves the inclusion of CDKN2A/B homozygous deletion as a marker. This genetic anomaly allows for the classification of the IDH-mutant astrocytoma as CNS WHO grade 4, regardless of histological features such as microvascular proliferation or necrosis [39].

#### Brief Genetic Overview of Relevance of CDKN2A/B Homozygous Deletion as a Marker

The CDKN2A and CDKN2B genes are critical in cell-cycle regulation, acting as tumor suppressors by encoding proteins that prevent unchecked cell proliferation, a hallmark of cancer [40]. Cyclin-dependent kinase inhibitor 2A (CDKN2A) is a gene located at chromosome 9, band p21.3. p14ARF and p15INK4b are proteins encoded by the CDKN2A and CDKN2B genes, respectively, and play crucial roles in cell-cycle regulation and tumor suppression [41,42].

p14ARF (alternate reading frame protein) functions primarily by activating the p53 tumor suppressor pathway, crucial for inducing cell-cycle arrest and apoptosis in response to oncogenic stress [37]. It achieves this by inhibiting mouse double minute 2 homolog (MDM2), a protein that targets p53 for degradation [37]. By binding to MDM2, p14ARF prevents the degradation of p53, allowing it to accumulate and activate genes that lead to apoptosis or cell-cycle arrest, thus preventing damaged cells from proliferating and potentially forming tumors [42].

On the other hand, p15INK4b is involved in regulating the cell cycle at the G1 checkpoint by inhibiting the activity of cyclin-dependent kinases CDK4 and CDK6 [43]. p15INK4b prevents the phosphorylation of the retinoblastoma protein (RB), maintaining RB in its active state, which binds to and sequesters E2F transcription factors, thus blocking the transition from G1 to S phase of the cell cycle [41,44,45,46].

Both proteins are vital in maintaining cellular integrity; mutations or deletions in the genes encoding these proteins can lead to uncontrolled cell division and contribute to the progression of various cancers [42,47]. Molecular insights are pivotal in exploiting specific weaknesses in a tumor’s genetic profile [48].

### 3.4. Imaging

The retention of functional CDKN2A and CDKN2B genes contributes to a more controlled cell cycle, and typically correlates with a less aggressive tumor behavior.As such, the slow-growing nature of CNS WHO grade 2 or 3 IDH-mutant astrocytomas, coupled with the brain’s ability to adapt and compensate, can make their presence less apparent until symptoms become more pronounced or the tumor is incidentally discovered during medical evaluation for other reasons, such as epilepsy [48].

On CT scans, IDH-mutant astrocytomas of CNS WHO grade 2 usually present as poorly defined, homogeneous, low-density masses without contrast enhancement [49]. As the tumor grade increases, features such as midline deviation, extensive edema (including peritumoral edema), contrast enhancement, and central hypodensity due to necrosis become more pronounced [49]. These changes reflect the progression of the tumor and the associated increase in aggressiveness and disruption of surrounding brain tissue. On CT scans, peritumoral edema can appear as areas of low density surrounding the tumor mass [50]. Peritumoral edema is an important radiological feature to identify, as it provides valuable information about the extent of tumor involvement and its impact on surrounding brain structures.

**Figure 1:**
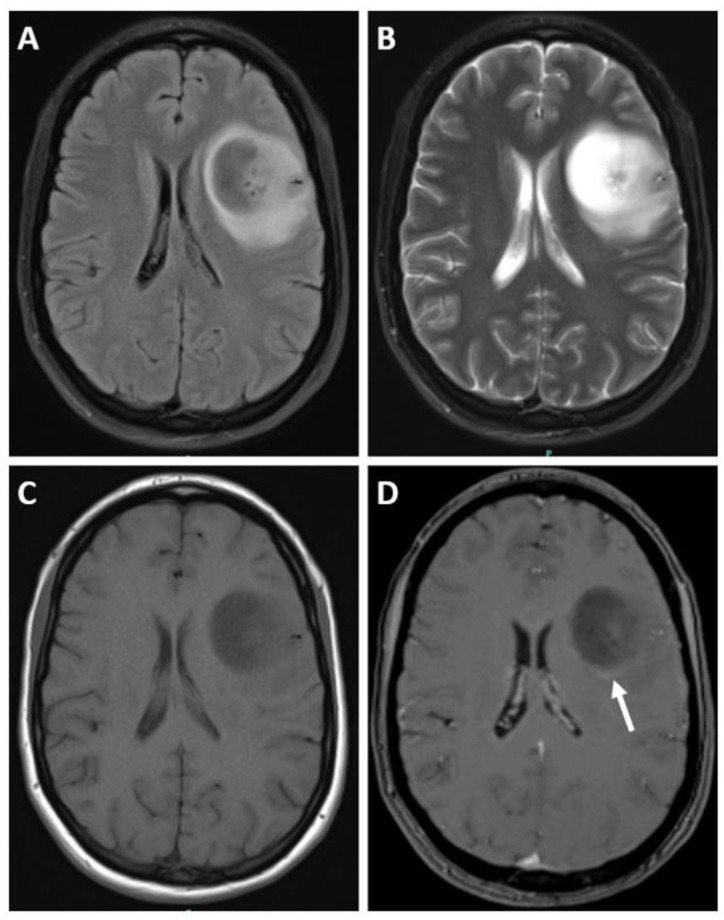
MR images derived from a 31 year old female with astrocytoma, Isocitrate dehydrogenase (IDH)-mutant, harboring a canonical IDH-1 mutation as well as alpha-thalassemia/mental retardation, X-linked (ATRX), and tumor protein p53 mutation. There was no cyclin-dependent kinase inhibitor 2A/B (CDKN2A/B) homozygous deletion. No increased mitotic activity was evident on histopathology; hence, it was classified as CNS WHO grade 2 IDH-mutant astrocytoma. (**A**) Axial T2-weighted fluid-attenuated inversion recovery (FLAIR) and (**B**) axial T2-weighted axial MRI scans demonstrate the presence of the T2-FLAIR mismatch sign, suggestive of WHO grade 2 and 3 IDH-mutant astrocytomas. (**C**) Axial T1-weighted image and (**D**) axial postcontrast T1-weighted fat-suppressed images demonstrate subtle peripheral enhancement (arrow). Note that the presence of contrast is uncommon in grade 2 IDH-mutant astrocytomas.

**Figure 2:**
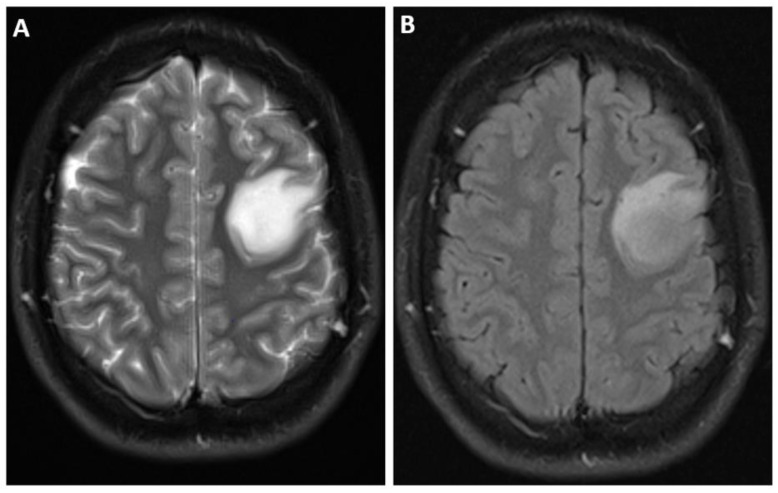
MR images derived from a 21 year old man with astrocytoma, isocitrate dehydrogenase (IDH)-mutant, WHO grade 2, harboring a canonical IDH1 mutation as well as loss of alpha-thalassemia/mental retardation, X-linked (ATRX), and tumor protein p53 mutation. No increased mitotic activity was evident on histopathology. There was nocyclin-dependent kinase inhibitor 2A/B (CDKN2A/B) homozygous deletion. (**A**) Axial T2-weighted fluid-attenuated inversion recovery (FLAIR) and (**B**) axial T2-weighted axial MRI images demonstrate left frontal lobe mass. T2-FLAIR mismatch sign is less conspicuous in this case.

On MRI, IDH-mutant astrocytomas exhibit certain characteristic features. These include T1 hypointensity and T2 hyperintensity, along with enlargement and distortion of involved areas [3,12]. Grade 2 IDH-mutant astrocytomas typically exhibit homogeneous T1-hypointense and T2-hyperintense masses without enhancement [51]. The T2- FLAIR mismatch sign, when seen, is highly suggestive of grade 2 and grade 3 IDH-mutant astrocytoma (Figure 1 and Figure 2) [52,53,54]. Signal characteristics of the T2-FLAIR mismatch sign include homogenous hyperintensity on T2-weighted images with a central hypointense signal and a thin rim of residual hyperintensity on fluid-attenuated inversion recovery (FLAIR) images. However, it is important to note that this sign may not be equally sensitive, and has been reported to result in false-positive findings [55].

Contrast enhancement, indicating increased vascularity, is uncommon in grade 2 tumors, but becomes more frequent in grade 3 and grade 4 tumors [48]. As the tumor grade increases, features such as T2 heterogeneity and elevated maximum relative cerebral blood volume (CBV) on perfusion imaging, in addition to contrast enhancement, become more prominent [48,56]. Rim enhancement around central necrosis can be observed in WHO grade 4 tumors [29]. Moreover, higher-grade lesions typically display more extensive surrounding edema On MRI scans, peritumoral infiltrative edema appears as areas of increased signal intensity surrounding the tumor on T2-weighted and FLAIR sequences. These areas typically appear brighter than normal brain tissue [57].

## 4. Oligodendrioglioma, IDH-Mutant and 1p/19q-Codeleted

### 4.1. Epidemiology and Localization

Both IDH-mutant astrocytomas and oligodendrogliomas share similarities in their epidemiology. They predominantly affect adults, with a typical age range of onset in the third to fifth decades of life [3]. Furthermore, both IDH-mutant astrocytomas and oligodendrogliomas exhibit a preference for the frontal lobes of the brain [3,14,58,59]. This shared anatomical predilection suggests potential commonalities in the microenvironment or cellular factors that contribute to tumorigenesis in this region [60,61,62]. Despite their distinct histopathological and genetic features, these similarities underscore overlapping characteristics in the clinical presentation and localization of these primary brain tumors [3].

### 4.2. Brief Genetic Overview

Oligodendrogliomas are characterized by a distinctive molecular profile that significantly influences their diagnosis, treatment, and prognosis [48,63]. Central to their molecular identity is the co-deletion of chromosome arms 1p and 19q, a predictor of a favorable response to chemotherapy and radiation, as well as a positive overall prognosis [63]. The co-deletion of chromosome arms 1p and 19q in oligodendrogliomas results in the loss of several genes that might be involved in DNA repair processes [64]. When these genes are missing, the tumor cells have a reduced ability to repair DNA damage caused by radiation or chemotherapy. As a result, these treatments may be more effective because the damaged tumor cells are less likely to survive and continue dividing [65].

Oligodendrogliomas are distinguished not only by their typical 1p/19q co-deletion and IDH mutations, but also by mutations in other specific genesleading to alterations in CIC (Capicua Transcriptional Repressor) and FUBP1 (Far Upstream Element Binding Protein 1) [66,67]. CIC is a transcriptional repressor that is involved in the regulation of gene expression within the MAPK signaling pathway [63,68]. Mutations in the CIC gene lead to a loss of function of this repressor. This eventually results in deregulated gene expression [68].

Oligodendrogliomas, unlike astrocytomas, rarely exhibit mutations in ATRX [63]. However, mutations in the telomerase reverse transcriptase (TERT) promotor are common [36]. While TERT promoter mutations generally enhance the expression of telomerase, helping tumor cells to maintain telomere length and survive longer, the presence of these mutations in the context of other vulnerabilities (like 1p/19q co-deletion and IDH mutation) might still contribute to a context where the overall DNA repair capabilities are compromised, rendering these cells more sensitive to treatments that cause DNA damage [7,37]. The presence of these molecular alterations—1p/19q co-deletion, IDH mutation, and TERT-promoter mutation—provides a robust framework for the classification of oligodendrogliomas [36,69].

Oligodendrogliomas are often classified into CNS WHO grades 2 or 3, primarily based on histological criteria, with additional consideration of CDKN2A/B homozygous deletion, which may serve as a molecular marker for assigning CNS WHO grade 3 status in certain instances [11,70].

### 4.3. Clinical Features

Oligodendroglioma, like IDH-mutant astrocytoma, could be described as insidious. The tumor might slowly grow without causing any noticeable symptoms until it has reached a significant size or has started to affect brain function [3]. The clinical presentation can vary significantly, but often includes seizures, which are the most common initial symptom due to the tumor’s frequent cortical involvement [48]. Other symptoms might include headaches, which can range from mild to severe depending on the tumor’s size and location, as well as personality changes and cognitive impairments when the frontal lobes are affected [63].

### 4.4. Imaging

**Figure 3:**
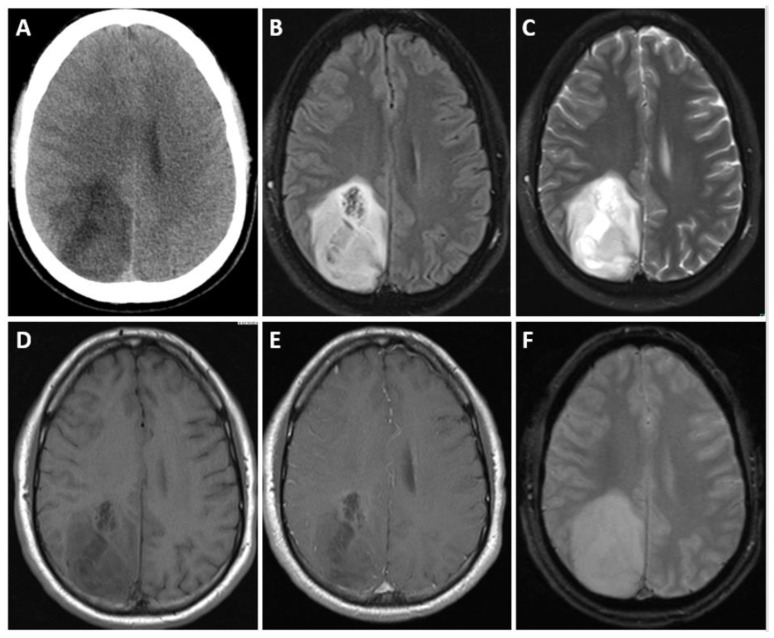
Axial unenhanced CT head (**A**), axial T2-weighted fluid-attenuated inversion recovery (FLAIR) (**B**), axial T2-weighted (**C**), axial T1-weighted (**D**), axial postcontrast T1-weighted (**E**), and multi-echo gradient recalled echo (GRE) (**F**) images in a 28 year old man with oligodendroglioma, isocitrate dehydrogenase (IDH)-mutant, and 1p/19q codeletion. Images demonstrate a heterogeneous right parieto-occipital region mass. This mass has indistinct margins and subtle contrast enhancement. Note: no calcification or hemorrhage on GRE or CT.

**Figure 4:**
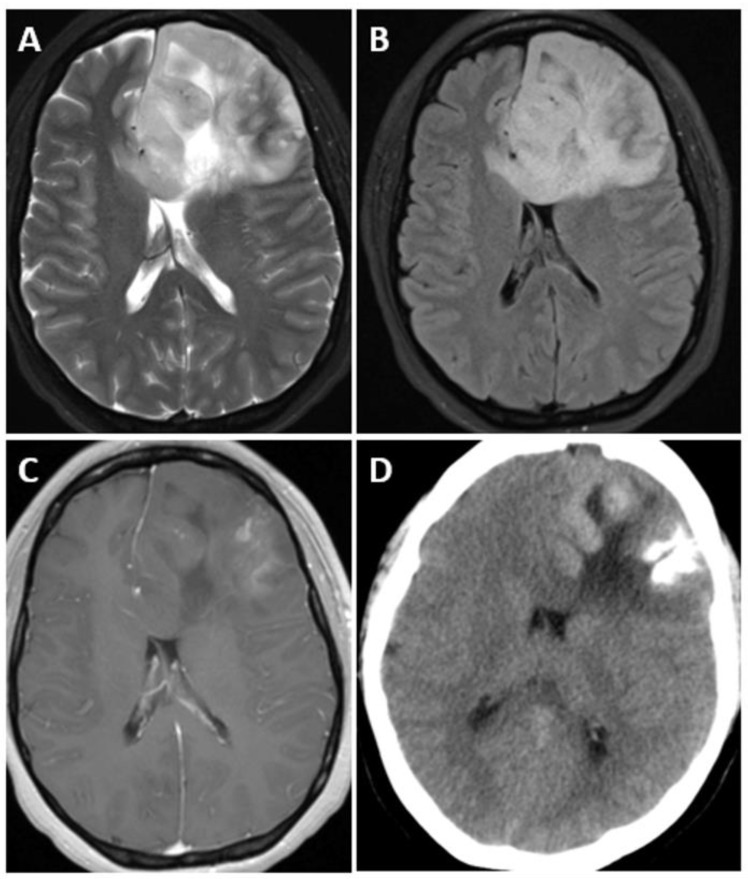
Axial T2-weighted (**A**), axial T2-weighted fluid-attenuated inversion recovery (FLAIR) (**B**), axial T1-weighted post-contrast(**C**), and unenhanced CT (**D**) through the level of the lateral ventricles in a 39 year old woman with oligodendroglioma, isocitrate dehydrogenase (IDH)-mutant and 1p/19q codeletion. Images demonstrate a heterogeneous left frontal lobe mass extending towards the right frontal lobe via the genu and proximal body of the corpus callosum. The mass demonstrates heterogeneous contrast enhancement and coarse calcifications.

On CT scans, IDH-mutant and 1p/19q-codeleted oligodendrogliomas commonly display as areas of decreased density (hypodense) or similar density (isodense) compared to surrounding brain tissue [59]. These lesions are often situated in the cortex and the underlying white matter of the brain. While the presence of calcifications is a frequent finding, it is important to note that calcifications alone are not sufficient for a definitive diagnosis of oligodendroglioma [59]. Additionally, some oligodendrogliomas may exhibit intratumoral hemorrhages or areas of cystic degeneration, further adding to the complexity of their appearance on imaging (Figure 3 and Figure 4) [51,59,71].

On MRI scans, oligodendrogliomas often appear as masses with low signal intensity on T1-weighted images and high signal intensity on T2-weighted images [59]. However, their margins may not be well-defined, leading to a blurred appearance [12]. Additionally, the signal intensities within the tumor can be heterogeneous, indicating variations in tissue composition and density [51,59,71].

When gadolinium-based contrast agents are administered, oligodendrogliomas may exhibit enhancement in some cases [51,59]. This enhancement, seen in less than 20% of grade 2 tumors but in over 70% of grade 3 tumors, is associated with increased microvascular proliferation within the tumor [71]. 

Comparing oligodendrogliomas to IDH-mutant diffuse astrocytomas reveals differences in vascular characteristics [59]. Oligodendrogliomas, in general, tend to demonstrate higher microvascularity and vascular heterogeneity [59]. These features can be observed on perfusion imaging techniques, indicating differences in blood flow patterns within the tumor vasculature [59].

Detecting IDH-mutant gliomas, including oligodendrogliomas, non-invasively is an area of active research. Magnetic resonance spectroscopy (MRS) is a specialized imaging technique that provides information about the chemical composition of tissues based on the signals emitted by atoms in different molecules [48,59,72]. In the context of gliomas, MRS can detect specific metabolic markers associated with tumor cells. In IDH-mutant gliomas, including oligodendrogliomas, there is a characteristic alteration in cellular metabolism that leads to the accumulation of a metabolite called 2-hydroxyglutarate (2-HG) [72,73]. This metabolite serves as a potential biomarker for the presence of IDH mutations in gliomas [73].

MRS allows researchers and clinicians to non-invasively measure the levels of 2-HG within the tumor tissue [73]. By analyzing the spectroscopic data obtained from MRS scans, it is theoretically possible to detect elevated levels of 2-HG, which indicate the presence of IDH mutations in the glioma cells [74]. However, implementing MRS for the detection of IDH-mutant gliomas in clinical practice presents several challenges [72]. The technique requires specialized equipment and expertise to acquire and interpret the spectroscopic data accurately. Additionally, factors such as tumor heterogeneity and variations in imaging protocols can affect the reliability of MRS measurements [48]. Its utility is also currently constrained by sensitivity and specificity issues. These limitations mean that MRS cannot fully replace the need for molecular diagnostics, which offer more precise and accurate information about tumor genetics and classification [74].

## 5. Glioblastoma, IDH-Wildtype

### 5.1. Update

The 2021 update to the WHO classification of brain tumors has refined the criteria for diagnosing glioblastoma, placing greater emphasis on molecular features over traditional histopathological signs [3]. In this updated classification, a glioblastoma is now defined not only by the presence of IDH-wildtype status, which indicates the absence of mutations in IDH1 or IDH2 genes, but also by the presence of one or more of the following features: microvascular proliferation, necrosis, TERT-promoter mutation, EGFR gene amplification, or +7/−10 chromosome copy number changes. This shift reflects an increased understanding of the molecular drivers behind glioblastoma’s aggressive behavior [17,75].

#### Brief Genetic Overview of Relevance of Molecular Markers

Glioblastomas are notoriously heterogeneous, with genetic and phenotypic variation, leading to significant therapeutic challenges [76]. Some of the alterations noted above portend a more aggressive course, and include TERT-promoter mutation, which allows cancer cells to bypass normal cellular aging and continue dividing indefinitely [36]. EGFR gene amplification results in the overexpression of the epidermal growth factor receptor, enhancing multiple downstream signaling pathways that promote cell proliferation, survival, and angiogenesis, characteristics central to the aggressive nature of glioblastoma [77,78]. Furthermore, chromosomal changes, specifically the gain of chromosome 7 and the loss of chromosome 10, introduce additional layers of complexity [79]. The gain of chromosome 7 often includes the EGFR gene, enhancing its oncogenic effects, while the loss of chromosome 10 frequently impacts the phosphatase and tensin homolong (PTEN) tumor-suppressor gene, leading to unregulated activation of growth pathways [17,75,79].

### 5.2. Clinical Features and Localization

Clinically, glioblastomas typically present with symptoms that reflect their fast growth, location, and high degree of malignancy [80]. Common symptoms include persistent headaches, nausea, and vomiting, which often are worse in the morning [81]. These symptoms are usually a result of increased intracranial pressure caused by the tumor mass [82]. Seizures can be an initial symptom due to the involvement of the tumor in the cerebral cortex. Depending on the tumor’s location in the brain, patients may experience a range of neurological deficits, such as weakness on one side of the body, speech difficulties, and vision problems [83]. Cognitive and personality changes are also sometimes observed, affecting the patient’s behavior and cognitive functions [3]. The rapid deterioration in neurological function is a hallmark of the disease, often leading to diagnosis at an advanced stage [83].

### 5.3. Imaging

**Figure 5:**
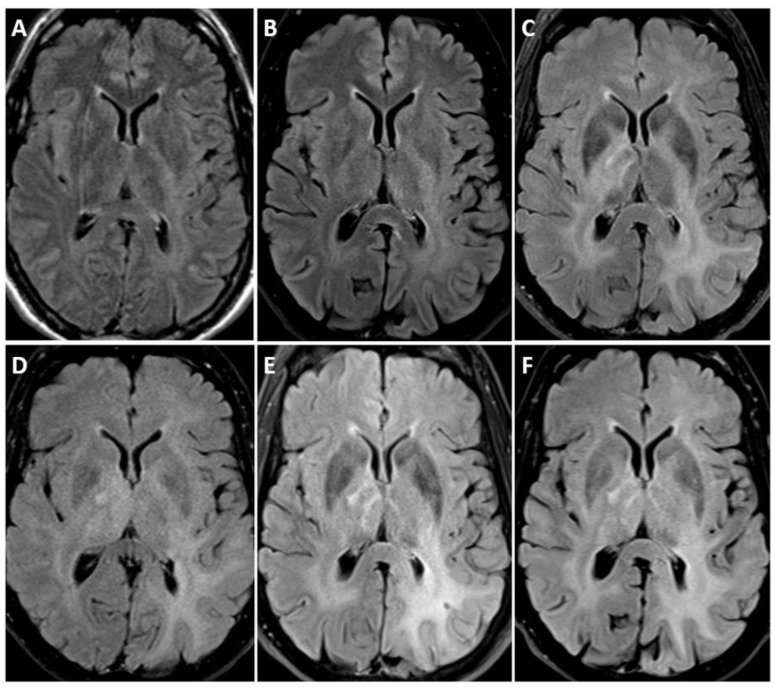
Imaging over the span of 10 years in a 71 year old man, demonstrating gradual progressive infiltrative changes diffusely throughout the left greater than right cerebral hemispheres, as depicted by T2/FLAIR hyperintensity. Axial T2-weighted fluid-attenuated inversion recovery (FLAIR) image at baseline (**A**), axial T2-weighted FLAIR image two years after baseline (**B**), axial T2-weighted FLAIR image four years after baseline (**C**), axial T2-weighted FLAIR image six years after baseline (**D**), axial T2 weighted FLAIR image obtained seven years after baseline and after left parietal biopsy (**E**), and axial T2 weighted FLAIR image obtained nine years after baseline and two years after biopsy (**F**). Histopathology showed no necrosis or microvascular proliferation. Immunohistochemistry was negative for isocitrate dehydrogenase (IDH) mutation, and next-generation sequencing identified an isolated reverse transcriptase telomerase (TERT)-promoter mutation, meeting 2021 CNS WHO criteria for glioblastoma (CNS WHO grade 4).

**Figure 6:**
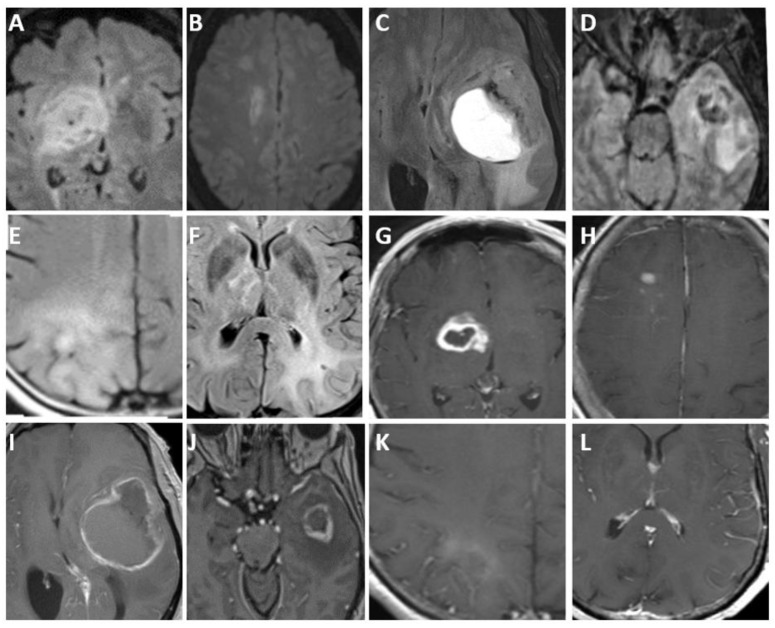
Spectrum of imaging found in five patients with WHO grade 4 glioblastoma, isocitrate dehydrogenase (IDH)-wildtype. Figures (**A**–**F**) are T2-weighted FLAIR, and (**G**–**L**) are post-contrast T1-weighted images. Figure components (**A**,**B**,**G**,**H**) are derived from a 74 year old woman with IDH-wildtype, +7/−10 chromosomal copy number changes. Images demonstrate a lesion centered in the right basal ganglia/ thalamocapsular region with ring enhancement (**G**), surrounding tumor infiltration, and vasogenic edema (**A**). Note the additional lesions with enhancement in the right frontal lobe (**H**). Figure components (**C**,**I**) are derived from a 55 year old man with left frontal IDH-wildtype glioblastoma, harboringtelomerase reverse transcriptase (TERT)-promoter mutation, epidermal growth factor receptor (EGFR) amplification, and +7/−10 chromosomal copy-number changes. Figure components (**D**,**J**) are derived from a 72 year old woman with left temporal IDH-wildtype glioblastoma, harboring TERT-promoter mutation, EGFR amplification, and +7/−10 chromosomal copy-number changes. Figure components (**E**,**K**) are derived from a 66 year old woman with IDH-wildtype glioblastoma with TERT-promoter mutation, demonstrating intrinsic T1 hyperintense signal (**K**), without enhancement. Note the presence of the surrounding FLAIR hyperintense signal (**E**). Figure components (**F**,**L**), derived from a 72 year old man originally diagnosed with “low-grade” glioma, but IDH-wildtype with TERT-promoter mutation, show infiltrative FLAIR signal (**F**) and no associated enhancement (**L**).

Glioblastomas, known for their aggressive behavior, present a spectrum of imaging features (Figure 5 and Figure 6) [84]. These tumors can infiltrate all areas of the brain, extending along white matter tracts and the corpus callosum to involve the contralateral hemisphere. Imaging manifestations of glioblastomas are inherently diverse, typically showcasing irregular ring enhancement around central necrosis, surrounding white matter tumor infiltration, and vasogenic edema [57]. The tumor typically has enhancing and non-enhancing components [85]. The rim of enhancing tissue is hypercellular and exhibits high cell turnover; on imaging, this is depicted by diffusion restriction, contrast enhancement, and high relative cerebral blood volume on perfusion. Patchy or no enhancement may also occur [85,86,87]. Moreover, glioblastomas have the capability to spread into adjacent lobes and deeply infiltrate the brainstem [88].

Despite the limitations of MRI in distinguishing morphological subtypes, subtle variations contribute to their overall diversity on imaging. For instance, giant cell glioblastomas and gliosarcomas can present with more circumscribed lesions located subcortically, resembling metastasis or meningiomas [89,90,91,92].

Moreover, assessing the extent of the tumor via imaging also poses challenges due to the presence of infiltrating tumor cells identified several centimeters from the tumor epicenter, even in the absence of T2/FLAIR changes, a likely site of recurrence [57,93,94].

Additionally, although less frequent, multifocal glioblastomas appear radiographically as multiple enhancing lesions on a background of T2-weighted signal changes, indicating a clear pathological connection [95,96]. Multicentric glioblastomas have also been described which can emerge in different lobes or hemispheres without a discernible connection [95,96,97].

## 6. Reiterated Update: Age Demographics in Glioma Classification

As mentioned above, in the 2021 WHO classification, gliomas are categorized into “adult-type” and “pediatric-type” based on age demographics, recognizing some overlap [3]. Age-related variations in glioma presentation are recognized, highlighting the importance of tailored diagnostic approaches for each patient population [22]. For example, for individuals under 55 or with a history of lower-grade glioma, negative IDH1 staining prompts DNA sequencing for rare IDH1 or IDH2 mutations [14]. The absence of IDH mutations leads to the classification of glioblastoma as an IDH-wildtype. Midline tumors require testing for the H3 p.K28M (K27M) mutation, while hemispheric tumors, especially in younger patients, necessitate examination for H3 G34-mutant diffuse hemispheric gliomas [98]. Also, it is important to acknowledge overlap, i.e., adult-type gliomas mainly affect adults, but may also occur in children, whereas pediatric-type tumors, commonly found in children, may occasionally present in adults [14].

### Pediatric-Type Diffuse High-Grade Glioma

This family includes Diffuse midline glioma, H3, K27-altered, Diffuse hemispheric glioma, H3 G34-mutant, Diffuse pediatric-type high-grade glioma, H3-wildtype and IDH-wildtype, and Infant-type hemispheric glioma [3].

## 7. Diffuse Midline Glioma, H3 K27-Altered

### 7.1. Epidemiology and Localization

Diffuse midline glioma, H3 K27-altered, represents a distinct category of brain tumors characterized by their aggressive nature and poor prognosis [20]. These tumors primarily affect children and young adults, although they can occur at any age [99]. They are most commonly located in the brainstem, thalamus, and spinal cord—regions critical for vital functions—which complicates treatment efforts [20].

### 7.2. Clinical Features

These aggressive brain tumors can have a rapid and severe onset due to their critical locations within the central nervous system. Most patients present with symptoms characterized by a classic triad: cranial nerve palsy, long tract signs, and ataxia [100]. Specifically, studies suggest that about 82% of patients experience cranial nerve palsy, which may manifest as facial weakness, double vision, or difficulty swallowing [101]. Long tract signs, including pyramidal tract impairment such as weakness, reflex abnormalities, and increased muscle tone, are observed in approximately 51% of cases [100]. Ataxia, which involves a lack of voluntary coordination of muscle movements, affects about 62% of these patients [101].

On the other hand, thalamic lesions commonly present with symptoms related to intracranial hypertension and motor or sensory deficits [101,102]. The increased intracranial pressure can lead to headaches, nausea, vomiting, blurred vision, and altered mental status [102]. Motor or sensory deficits due to the tumor’s impact on the thalamic region, a critical area for sensory and motor signal relay, can result in varying degrees of paralysis or abnormal sensations [103].

Given their location in the brainstem and other midline structures, these tumors often impact the function of surrounding nervous tissue, leading to progressive and multifaceted symptoms. Seizures are less common in diffuse midline gliomas compared to other brain tumors due to their deep location away from the cerebral cortex [102].

### 7.3. Brief Genetic Overview

Chromatin is made up of nucleosomes, which are histone octamers wrapped by DNA. It helps regulate key cellular processes like transcription, replication, DNA repair, and genomic stability. This regulation is achieved through post-translational modifications of DNA and histones [104]. In humans, different histone H3 variants exist. H3.3 proteins are expressed continuously, whereas H3.1 and H3.2 are only expressed during the S phase of the cell cycle [104]. H3.3 is encoded by two genes, H3F3A and H3F3B [104]. Diffuse Midline Glioma, H3 K27-altered, harbors a mutation primarily of the of the H3F3A gene, leading to amino acid substitution where a lysine side chain, the 27th residue, is typically replaced by methionine [105].

The presence of the H3 K27M mutation initiates a series of molecular events that significantly alter the epigenetic landscape of the affected cells. H3K27 can undergo mono-methylation (H3K27me1), di-methylation (H3K27me2), tri-methylation (H3K27me3), or acetylation (H3K27ac) [104]. This mutation results in a hypomethylated state of H3K27me2 and H3K27me3 across the genome [106]. In vitro, H3K27M appears to affect the enzymatic activity of EZH2, a part of the Polycomb Repressive Complex 2 (PRC2) that typically adds methyl groups to H3K27 [107]. This interference results in a substantial decrease in H3K27me3 levels and presumed transcription dysregulation with derepression, contributing to the abnormal gene expression patterns observed in these tumors [104,108,109].

Diffuse midline gliomas, H3 K27-altered can be classified into distinct subclassifications based on the histone H3 variant [110,111,112]. The first subtype encompasses H3.3 - mutant cases [112]. The H3.3 p.K28M (K27M)-mutant involves mutations in the H3F3A gene encoding the H3.3 histone variant, predominantly affecting children and typically found in vital brain regions like the brainstem and thalamus [112]. The H3.1 or H3.2 p.K28M (K27M)-mutant subtype includes mutations in the HIST1H3B or HIST1H3C genes; these might exhibit distinct biological behavior, occurring in the pons of younger patients [3,111,112]. The third subtype is characterized by H3-wildtype tumors with overexpression of EZHIP, which mimics the effects of the K27M mutation by inhibiting the PRC2 complex. This leads to similar epigenetic deregulation [107,108,113,114]. Lastly, the EGFR-mutant subtype is driven by mutations in the EGFR gene, and is associated with varying tumor behaviors compared to histone-mutated gliomas [115,116]. Thus, given recognition of alternative mechanisms, the name has been changed from H3 K27-mutant to H3 K27-altered.

### 7.4. Imaging

**Figure 7:**
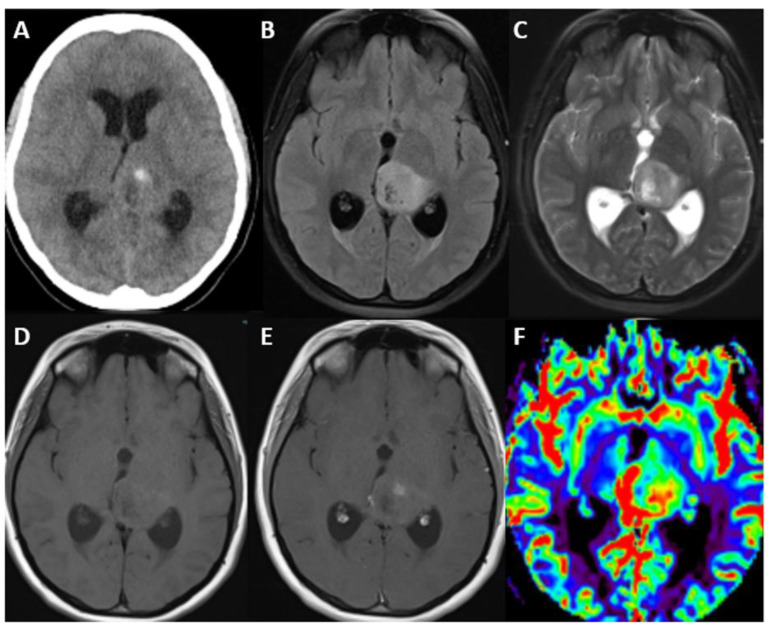
Axial unenhanced CT head (**A**), axial T2-weighted fluid-attenuated inversion recovery (FLAIR) (**B**), axial T2-weighted (**C**), axial T1-weighted (**D**), axial T1-weighted fat suppressed post-contrast (**E**), and cerebral blood volume (CBV) perfusion map (**F**) through the level of the thalamus in a 17 year old woman with diffuse midline glioma, H3K27-altered. Images illustrate an expansile, hyperintense left thalamic mass, more typical of adolescents/adults. This mass demonstrates heterogeneous contrast enhancement and increased perfusion. Histopathological diagnosis and grade were consistent with diffuse astrocytoma, WHO grade 2, by morphology. The patient had multiple recurrences with resections and radiation necrosis, eventually succumbing to the disease three years after presentation.

These tumors have variable imaging characteristics, but can appear on MRI as ill-defined, infiltrative masses primarily located within the brainstem, thalamus, or spinal cord, frequently with no enhancement [112]. The H3.1 subtype reportedly has an increased propensity to demonstrate edema and necrosis with ring enhancement [111]. On T1-weighted MRI sequences, they usually present as hypointense or isointense, whereas on T2-weighted and Fluid-Attenuated Inversion Recovery (FLAIR) sequences, they are hyperintense. Contrast enhancement in general can vary, but many diffuse midline gliomas show a patchy or heterogeneous pattern after the administration of gadolinium-based contrast agents, which may correlate with areas of higher tumor cell density or a disrupted blood–brain barrier (Figure 7) [117].

## 8. Diffuse Hemispheric Glioma, H3 G34-Mutant

In the 2021 WHO classification, a newly recognized tumor is Diffuse Hemispheric Glioma, H3 G34-mutant [118]. This tumor is characterized by mutations in H3F3A, leading to substitution at position G34, which influences both the biological behavior of the tumor and its response to therapy [111,119].

### 8.1. Clinical Features

Clinically, patients with H3 G34-mutant gliomas may present with symptoms related to increased intracranial pressure, such as headaches, nausea, and vomiting, or with seizures, focal neurological deficits, and cognitive or behavioral changes depending on the location of the tumor within the cerebral hemispheres [120].

### 8.2. Epidemiology and Localization

The epidemiology of diffuse hemispheric glioma with H3 G34 mutation shows a slight male predominance and a typically more aggressive course than other pediatric brain tumors [119]. This mutation is less common than the H3 K27M mutation found in midline gliomas, with specific genetic and clinical features that differentiate them from other glioma subtypes [121].

### 8.3. Brief Genetic Overview

Driving mutation here is a missense mutation of the H3F3A gene, eventually causing glycine 34 to be replaced by arginine or valine in the mature protein [121]. While G34 is not subject to methylation, this particular mutation leads to changes that inhibit binding of regulatory proteins and ultimately causes transcriptional reprograming [3,105]. Often, this mutation is found alongside alterations in Alpha Thalassemia/Mental Retardation Syndrome X-linked (ATRX) and mutations in TP53 [119].

### 8.4. Imaging

These lesions can present on MRI scans as contrast-enhancing tumors causing mass effect in cortical areas, commonly in the parietal or temporal lobe, with features that may include necrosis, cystic changes, hemorrhage, and calcifications [122]. They have been described as unifocal lesions with a propensity for ependymal and leptomeningeal contact [112,120].

## 9. Diffuse Pediatric-Type High-Grade Glioma, H3-Wildtype and IDH-Wildtype

Diffuse pediatric-type high-grade glioma H3-wildtype and IDH-wildtype is a heterogeneous group of tumors characterized by a combination of high-grade morphology, which can be either glial or primitive/undifferentiated [112].

### 9.1. Brief Genetic Overview

These tumors lack mutations in the IDH1 or IDH2 genes, and do not exhibit histone H3 alterations. Instead, they align with one of three pediatric high-grade glioma (pHGG) methylation groups: pHGG receptor tyrosine kinase 1 (RTK1), pHGG receptor tyrosine kinase 2 (RTK2), or pHGG V-Myc Avian Myelocytomatosis Viral Oncogene Neuroblastoma-Derived Homolog (MYCN) [112]. Alternatively, these tumors may be identified by the genetic alterations that are characteristic of these methylation groups, involving Platelet-Derived Growth Factor Receptor Alpha (PDGFRA) for the RTK1 group, EGFR for the RTK2 group, or MYCN for the MYCN group [112,123,124].

#### Relevance

The molecular pathology often involves the activation of RTKs like PDGFRA and EGFR, which trigger critical downstream signaling pathways including PI3K/AKT/mTOR and RAS/MAPK [21]. These pathways are essential for cell proliferation and survival, making them attractive targets for therapeutic intervention. For instance, the use of MEK inhibitors, which act on the MAPK pathway, has demonstrated potential in preclinical trials to curb tumor growth [125].

Furthermore, specific clinical scenarios, such as gliomas that develop after therapeutic radiation or those associated with germline mismatch repair deficiencies, often exhibit molecular characteristics, particularly the RTK1 subtype involving PDGFRA amplification [126,127].

### 9.2. Imaging

**Figure 8:**
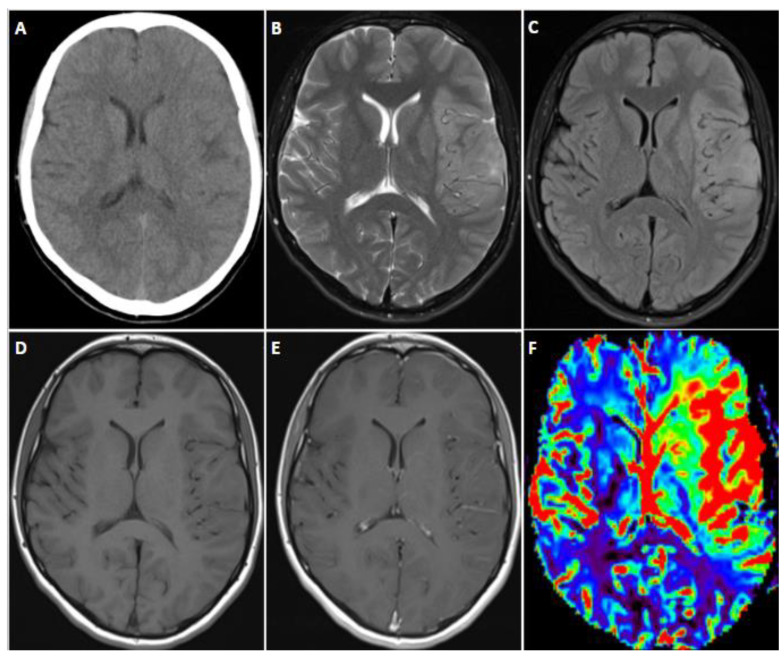
Axial unenhanced CT (**A**), axial T2- weighted (**B**), axial T2 fluid-attenuated inversion recovery (FLAIR) (**C**), axial T1-weighted (**D**), axial T1-weighted post-contrast (**E**), and cerebral blood volume map from dynamic susceptibility contrast (DSC) perfusion images (**F**) through the level of the lateral ventricles in a 22 year old female with diffuse hemispheric glioma, H3 G34-mutant and IDH-wildtype tumor, who presented with first-time seizure. The images demonstrate an infiltrative left frontal and left temporal operculum mass, as depicted by abnormal T2 FLAIR hyperintensity, with associated increased perfusion and no abnormal contrast enhancement. Lesion is unifocal with leptomeningeal contact at its lateral aspect. There was no diffusion abnormality. Note that this was initially misdiagnosed as acute encephalitis.

**Figure 9:**
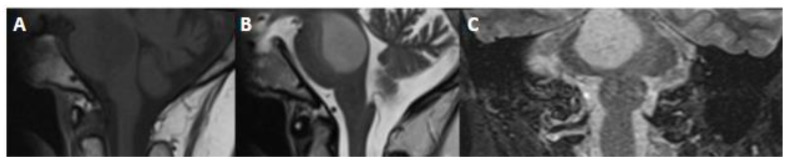
Sagittal T1-weighted (**A**), sagittal T2-weighted (**B**), and coronal T2-weighted short tau inversion recovery (STIR) cervical spine images (**C**) in a 32 year old woman with previously treated intracranial germinoma who presented with left sensorineural deficits. Images demonstrate an expansile, T1 hypointense and T2 hyperintense central pontine mass. This was diffuse pediatric-type high-grade glioma, H3-wildtype, receptor tyrosine kinase (RTK1) subtype, subclass C. RTK1 subtypes typically involve the supratentorial brain, with approximately 18% located in infratentorial/brainstem structures. Gliomas arising after therapeutic radiation, as in this patient, are predominantly of the RTK1 molecular subtype.

MRI characteristics typically resemble those of other high-grade gliomas, presenting as T2 hyperintense tumors with varying degrees of contrast enhancement and mass effect (Figure 8 and Figure 9) [120,124].

## 10. High-Grade Astrocytoma with Piloid Features

High-grade astrocytoma with piloid features (HGAP) represents a distinct category within the spectrum of brain tumors, newly recognized for its unique DNA methylation patterns that set it apart from other gliomas [3,128].

Epidemiologically, HGAP remains rare, and its exact incidence is still being delineated as the medical community gains a better understanding of its distinct molecular characteristics [3].

**Figure 10:**
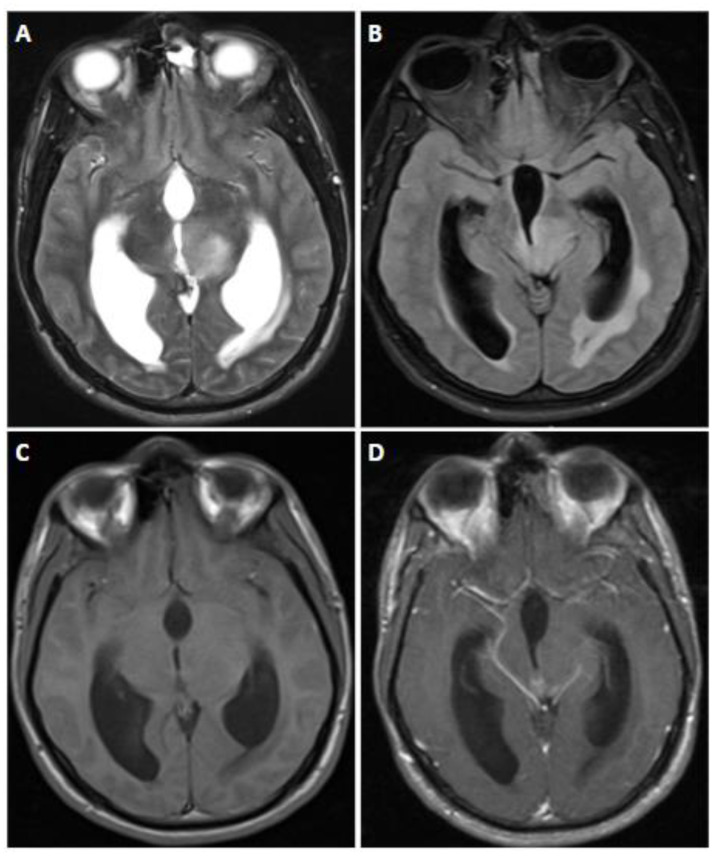
Axial T2-weighted (**A**), axial T2 fluid-attenuated inversion recovery (FLAIR) (**B**), axial T1-weighted (**C**), and axial T1-weighted post-contrast (**D**) images in 27 year old man with high-grade astrocytoma with piloid features (HGAP). Images demonstrate a heterogeneous T2 hyperintense mass centered in the left thalamus, extending into the tectal plate. There were some areas of heterogeneous enhancement. Local mass effect and obstruction at the level of the cerebral aqueduct resulted in upstream obstructive hydrocephalus. Molecular profiling showed isocitrate dehydrogenase (IDH) wildtype, alpha-thalassemia/mental retardation, X-linked (ATRX), Neurofibromin 1 (NF1) mutation, and cyclin-dependent kinase inhibitor 2A/B (CDKN2A/B) homozygous deletion. Collective molecular features suggest high-grade astrocytoma with piloid features (HGAP), according to WHO CNS5 tumor classification.

HGAP tumors can occur anywhere in the central nervous system (CNS), but they have a predilection for the cerebellum, where they can cause symptoms related to increased intracranial pressure, cerebellar dysfunction, or cranial nerve deficits, depending on their size and exact location [128,129] (Figure 10). They can be seen in the brain or spine, often midline or paramedian in location [130]. Imaging suggests a propensity for rim enhancement and central necrosis [129,130].

### Brief Genetic Overview

From a genetic standpoint, HGAP commonly exhibits alterations in genes associated with the mitogen-activated protein kinase (MAPK) pathway, which is pivotal in cell growth and differentiation [128,129]. Additionally, this tumor type frequently shows homozygous deletion of the CDKN2A/B genes, which are crucial tumor suppressors regulating the cell cycle [129]. Loss or mutation of ATRX, a gene involved in chromatin remodeling, is another hallmark of HGAP, contributing to its distinct molecular profile [129].

## 11. Discussion

The 2021 World Health Organization (WHO) classification of central nervous system (CNS) tumors has ushered in a paradigm shift. This update underscores the increasing importance of genetic and molecular markers in the management of gliomas.

This molecular approach allows for deeper insights into tumor behavior, enabling more accurate prognostication and guiding treatment decisions, thus allowing for the development of personalized treatment plans tailored to the specific genetic profile of each tumor. As insight increases, the aim would be to maximize therapeutic efficacy and minimize adverse effects, ultimately improving patient outcomes.

Furthermore, the 2021 WHO classification is expected to drive significant advancements in research by providing a clearer framework for studying tumor biology. This, in turn, will likely accelerate the translation of research findings into clinical applications, hopefully fostering the development of innovative treatments that improve survival rates and quality of life for patients.

Ultimately, the standardized terminology and classification criteria introduced by the WHO update promotes consistency in diagnosis and treatment across healthcare systems. This enhances the comparability of clinical trial results, supports global treatment guidelines, and fosters international collaboration in research. Aligning clinical practice with the latest scientific insights ensures that patients worldwide benefit from advanced diagnostic and therapeutic strategies.

The updates and some imaging findings are summarized in Table 1 and Table 2.

## 12. Conclusions

In conclusion, the 2021 WHO classification of CNS tumors represents a significant advancement in the field of medicine. By incorporating molecular diagnostics into the classification and grading of gliomas, the update enhances diagnostic precision and aligns clinical practice with the latest scientific insights. This integration of genetic and molecular data into routine diagnostic protocols is pivotal for accurate classification, prognosis, treatment, and follow-up strategies.

Radiologists and other healthcare professionals involved in the care of patients with CNS tumors must stay abreast of these updates to effectively contribute to multidisciplinary tumor boards and collaborate with peers in neuro-oncology, neurosurgery, radiation oncology, and neuropathology. The comprehensive understanding of molecular underpinnings provided by these updates facilitates personalized treatment approaches, ultimately improving patient outcomes through informed and precise imaging assessments.

Overall, the 2021 WHO classification marks a significant step forward in the understanding and management of CNS tumors. By further emphasizing molecular diagnostics, it promises to significantly advance the field, driving improvements in research, therapy, and global collaboration, ultimately leading to better patient outcomes. This review aims to simplify these changes and provide illustrative case examples to enhance understanding and application in clinical practice.

## Figures and Tables

**Table 1:** Adult-type diffuse gliomas, alterations, imaging characteristics, and updates from 2016 WHO classification of CNS tumors.

Adult-Type Diffuse Glioma	Some Common Genetic/Molecular Alterations	Interval Update	Typical Imaging Characteristics
Astrocytoma, IDH-mutant	IDH1, IDH2, ATRX, TP53	CDKN2A/B homozygous deletion designated grade 4, no IDH-wildtype	Homogenous, circumscribed, T2-FLAIR mismatch sign
Oligodendroglioma, IDH-mutant and 1p/19q-codeleted	IDH1, IDH2, 1p/19q, TERT Promotor, CIC, FUBP1	CDKN2A/B homozygous deletion indicates a CNS WHO grade 3	Heterogeneity, calcification, variable enhancement, possible increased rCBV
Glioblastoma, IDH-wildtype	IDH-wildtype, TERT promotor, +7/−10 chromosome copy number changes, EGFR	Only IDH-wildtype no IDH-mutant,	Inherent heterogeneity, heterogeneously enhancing, sometimes ring- enhancing around central necrosis, surrounding T2/FLAIR signal, edema and mass effect, increased rCBV, surrounding infiltration.
		IDH-wildtype astrocytic tumors with 1 of following: Microvascular proliferation or necrosis, TERT promotor mutation, or EGFR gene amplification, +7/−10 chromosome copy number changes.	

**Table 2:** Pediatric-type diffuse high-grade gliomas, alterations, imaging characteristics, and updates from 2016 WHO classification of CNS tumors.

Pediatric-Type Diffuse High-grade Gliomas	Some Common Alterations	Interval Update	Imaging Characteristics
Diffuse midline glioma, H3 K27-altered	H3 K27, TP53, ACVR, PDGFRA, EGFR, EZHIP	Name changed from H3K27-mutant,	Variable, ill-defined, infiltrative masses, in brainstem, thalamus, or spinal cord, frequently no enhancement, H3.1 subgroup-necrosis and edema likely.
		Subgroups recognized	
Diffuse hemispheric glioma, H3 G34-mutant	H3 G34, TP53, ATRX	Newly recognized	Unifocal, cortex, ependymal and/or leptomeningeal contact.
Diffuse pediatric-type high-grade glioma, H3-wildtype, and IDH-wildtype	No IDH or H3 mutations, PDGFRA, MYCN, EGFR	Newly recognized	Heterogeneous, akin to other high-grade gliomas.
Infant-type hemispheric glioma (not discussed in this review)	NRTK family, ALK, ROS, MET	Newly recognized	Large masses with frequent superficial involvement including leptomeninges.
